# Assessing Design Space for the Device-Circuit Codesign of Nonvolatile Memory-Based Compute-in-Memory Accelerators

**DOI:** 10.1021/acs.nanolett.4c05299

**Published:** 2025-01-13

**Authors:** Ashwin Sanjay Lele, Bo Zhang, Win-San Khwa, Meng-Fan Chang

**Affiliations:** † Corporate Research, TSMC, San Jose, California 95134, United States; ‡ Corporate Research, TSMC, Hsinchu, 300-094, Taiwan; § National Tsing Hua University, Hsinchu, 300-044, Taiwan

**Keywords:** compute-in-memory, nonvolatile memory, RRAM, PCM, MRAM

## Abstract

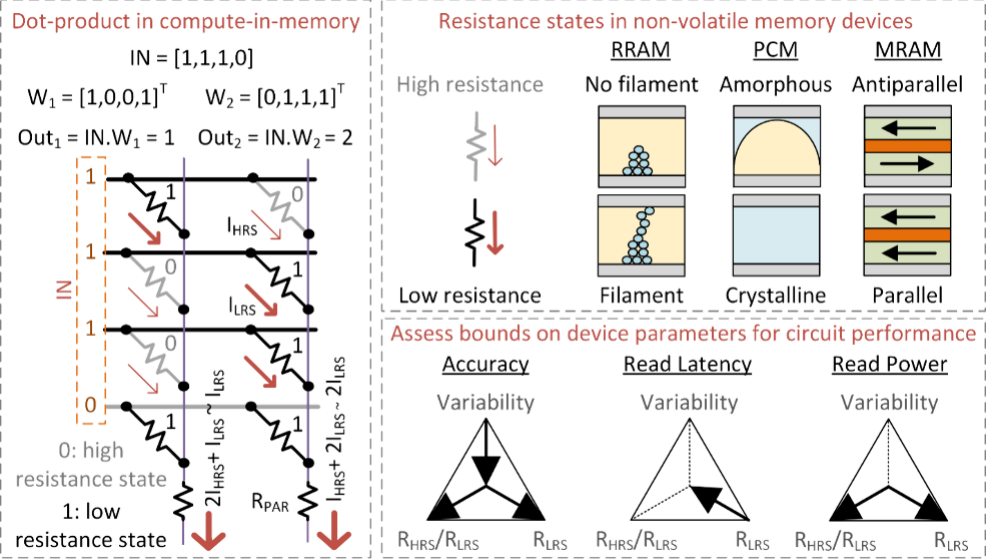

Unprecedented penetration of artificial intelligence (AI) algorithms has brought about rapid innovations in electronic hardware, including new memory devices. Nonvolatile memory (NVM) devices offer one such attractive alternative with ∼2× density and data retention after powering off. Compute-in-memory (CIM) architectures further improve energy efficiency by fusing the computation operations with AI model storage. Electronic characteristics of NVM devices, like resistance in the two resistance states, directly affect the circuit designers’ decisions and result in the varying performance of NVM-CIM chips. In this mini review, we assess the bounds on device resistances for accuracy and circuit performance to suggest recommendations to device engineers for frictionless device–circuit–system interactions. Furthermore, we review challenges in reliably programming NVM devices, followed by benchmarking recent NVM-CIM chips. Our literature review and analytical modeling reveal that a high resistance ratio and low variability are favored, and the resistance in a low resistance state is bound by accuracy and circuit performance constraints.

## Introduction

**Figure 1:**
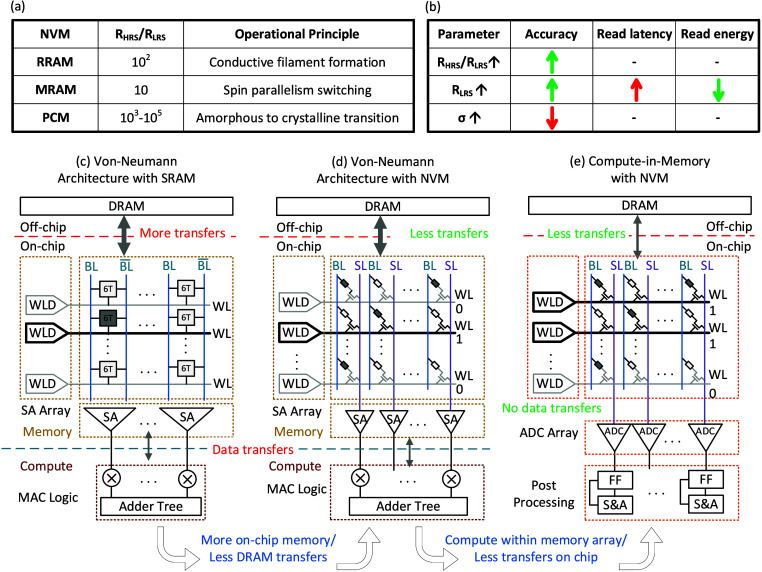
(a) Nonvolatile memory (NVM) devices with their operational principles and typical device characteristics.^8^ (b) Effect of device parameters on application-level performance metrics. (c–e) Circuit schematics for conventional von Neumann architecture, NVM as on-chip memory, and NVM-based CIM.

AI workloads require storage of models close to the computation units to avoid data movement to improve both energy efficiency and throughput.^1,2^ SRAM is conventionally used for on-chip storage because of its CMOS compatibility, reliable operation, and scaling to advanced technology nodes.^3^ SRAM is complemented by DRAM for larger and denser off-chip memory hosted on a separate die. The difference in fabrication process prevents DRAM integration on the CMOS flow, while a larger area of SRAM caused by a 6-transistors (6T) bitcell hinders large on-chip storage. Nonvolatile memory (NVM) devices offer advantages of density, like DRAM and CMOS, compatible with on-die integration, like SRAM.^4−6^ 1-Transistor-1-resistor (1T1R) bitcells of RRAM and MRAM demonstrate significant high density compared to 6T SRAM bitcells. Their memory operation stems from their physical characteristics like material crystallization in phase change memory (PCM), conductive filament formation in RRAM, and spin alignment in MRAM.^7^ This gives rise to distinct resistance states called the low resistance state (LRS) and high resistance state (HRS). These binary states offer distinguishable currents when sensed using a reading voltage (*V*_read_) and can be used to store 0/1. NVMs have been shown to be integrated within the CMOS process at various nodes, like 12, 22, and 40 nm for RRAM,^8−10^ 14, 22, and 18 nm for MRAM,^11−13^ and 14 and 40 nm for PCM.^14−16^ Additionally, they retain the stored data even when they are isolated from the power supply, resulting in additional energy savings in standby operations. Typical resistance characteristics and operational principles of popular NVMs are shown in Figure 1a.

Nevertheless, energy overheads of fetching and storing the data between memory and compute still continue if NVMs are used only for on-chip storage.^10^Figure 1c shows a conventional Von-Neumann architecture with an AI model stored in the memory with a physically separate compute unit carrying out MAC operations. Constant data movement between memory and compute causes significant energy and latency overhead.^17^ NVM storage improves memory density and may reduce transfers to and from DRAM (Figure 1d). Compute-in-memory (CIM) takes a more aggressive approach and merges a part of the MAC operation within memory array to reduce data movement even more for efficiency and speed. Figure 1e shows an example of an analog CIM array with NVM devices like RRAM storing the weights using HRS and LRS as bits 0/1, respectively. The wordline (WL) is driven by the input activation through a wordline driver (WLD) and the NVM device allows passage of current depending on the resistance state. Bit-wise multiplication happens within the NVM devices, whereas accumulation is carried out over the BL/SL. The sum of the currents represents the multiply and accumulation (MAC) result between activation and weight, and it is converted to a digital code for post-MAC processing by an analog-to-digital convertor (ADC). Without extra data movement and multirow access, the CIM macro usually exhibits high energy efficiency (tera-operations/sec/watt) and high compute density (tera-operations/sec/mm^2^).

Numerous material and device candidates have been proposed in recent years for NVM-CIM^18^ operations with different switching materials and electrodes,^7,19,20^ e.g., resistance in LRS may vary from 700 Ω to 930 MΩ for RRAM^20^ and 900 Ω to 6 MΩ for MRAM.^21^ However, the resistance ratio (*K* = *R*_HRS_/*R*_LRS_) remains relatively constant, as shown in Figure 1a. Different devices provide a large range of resistance values (LRS/HRS), write characteristics, and endurance performance.^20^ MAC operation in CIM is known to be affected by the resistance ratio (*K* = *R*_HRS_/*R*_LSR_), read current in LRS (*I*_LRS_ = *V*_read_/*R*_LRS_), and process-induced variability (σ)^22,23^ (Figure 1b). These parameters affect CIM accuracy, energy consumption, and compute latency at circuit level, and further, at system level.^24−26^ Therefore, early identification of device parameter design space given circuit/system specifications helps the material/device researchers to make design choices in the development of these resistive memory devices. In this mini review, we survey the recent literature to provide an analytical model on how device parameters affect circuit designs for CIM readout and suggest recommendations to the device and material engineering community for seamless device–circuit interactions. We focus on maintaining accuracy for readout and minimizing the energy-delay product for the CIM-array to identify bounds on the device parameters. Our modeling framework may be useful for early design decisions by materials, devices, and circuit engineers, while the summary of literature shows upcoming challenges and research trends that may push the viability of NVM-CIM for commercial applications.

## Device Parameter Design Space for NVM-CIM

**Figure 2:**
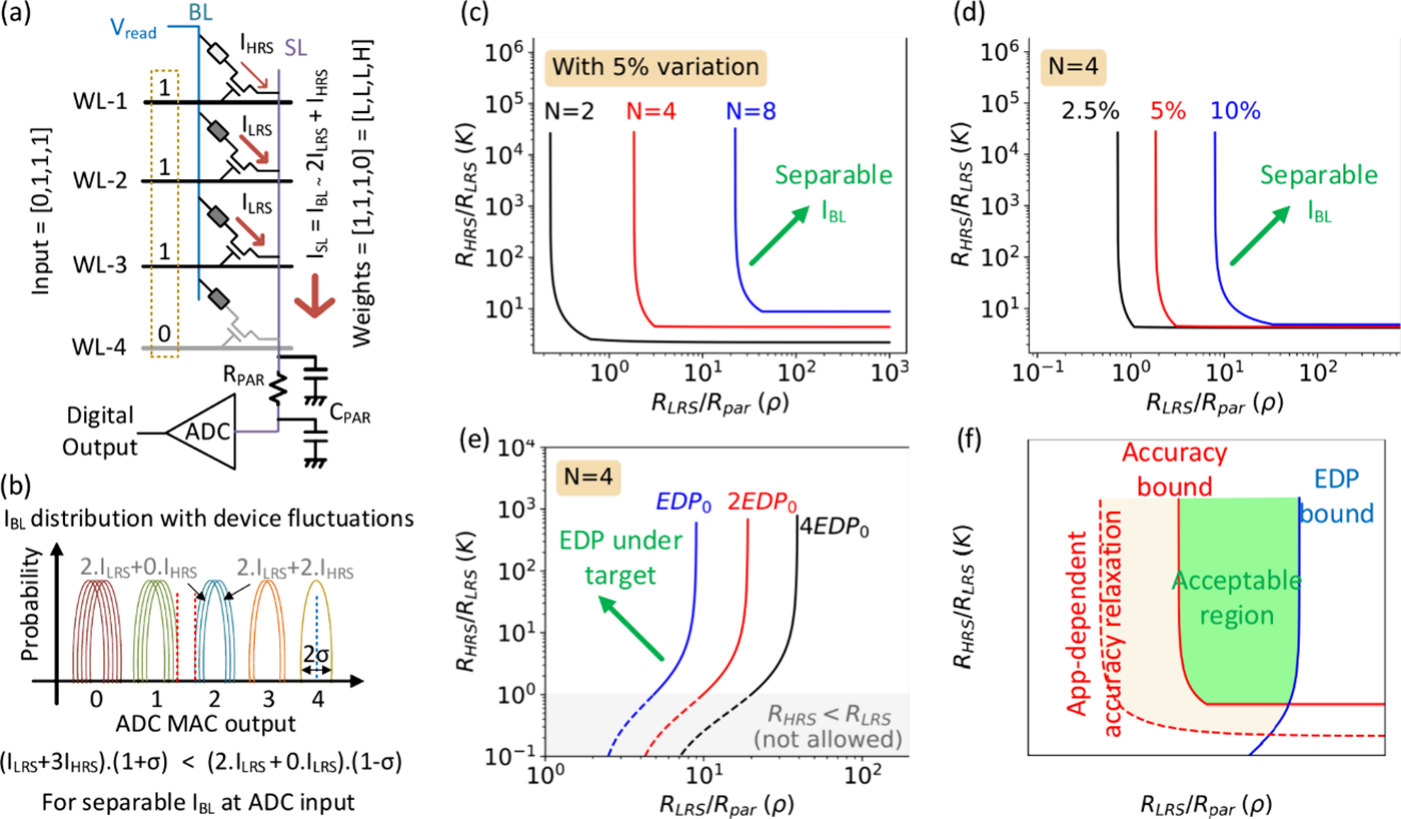
(a) CIM read path–circuit model (b) Distribution of bitline currents with expected ADC output (c, d) Acceptable resistance values for *I*_BL_ separable readout. (e) Constraints on resistance values caused by EDP budget. (f) Design space for the device determined by application-level specifications.

CIM arrays involve preprogramming of devices (cells) for storing the binarized AI model into HRS/LRS before inference computation begins. Devices with more than two resistance states have also been demonstrated for RRAM^27^ and PCM.^14^ However, their stability, technological maturity, and circuit accuracy at readout lags behind binarized states.^28^ Therefore, we focus on a two-resistance state analysis in this section. Computation involves applying input activations to the WLs of the NVM array and reading out BL/SL currents (*I*_BL_, *I*_SL_) for MAC computation. A typical CIM configuration is shown in Figure 2a, where the activation (input) vector in the form of binary numbers is applied at the WL. When the input is 1 (WL-1,2,3), the access transistor in the cell is on, allowing current to flow from BL to SL, whereas when input is 0, the access transistor prevents the current flow across the cell. As cell-2 and cell-3 are at the LRS, a relatively high current (*I*_LRS_) flows through them. As for the HRS cell (cell-1), a small current (*I*_HRS_) flows. Assume that the *I*_LRS_ is considerably higher than *I*_HRS_, then the total current on SL is *I*_SL_ = *I*_HRS_ + 2*I*_LRS_ ≈ 2*I*_LRS_, which is proportional to the MAC result. The ADC at the end of the source line senses the current and provides a digital output corresponding to the analog MAC value.

### Preserving Computation Accuracy with Separable *I*_BL_

For the actual device, *I*_HRS_ is non-negligible compared to *I*_LRS_. It adds up to *I*_BL_ and hurts the proportionality of the MAC computation.^29^ Assume an input vector of size *N* drives *N* WLs (through WLD) simultaneously, the possible values of MAC are 0, 1, ..., and *N*. However, multiple expected *I*_SL_ can correspond to one single MAC value depending on the number of HRS cells turned on. Moreover, due to device variation, *I*_SL_ also fluctuates around the expected current value. Figure 2b shows the probability distribution of the MAC result with the *I*_HRS_-effect and the current variation, e.g., for *N* = 4, 2 LRS cells allow passage of *I*_LRS_, while the remaining 2 HRS cells causing *I*_HRS_, this case corresponds to MAC result being “2”. Similarly, the case with 2 LRS passing *I*_LRS_ and 2 cells in off state also corresponds to MAC result being “2”. Each of these two cases has its own variation distribution, and both contribute to the MAC = 2 distribution. For distinguishability at ADC input, the distributions across different MAC result need to be separable as shown, i.e., the highest value of *I*_BL_ for digital output “*n*” needs to be smaller than lowest value of *I*_BL_ for digital output “*n* + 1”. This provides the preliminary condition for the MAC computation to be accurate, as shown in the equation from Figure 2b. It is important to note that separability of *I*_BL_ does not guarantee accurate readout as it also depends upon the ADC design and read margin of ADC. However, it provides the necessary condition for ADC to be able to distinguish between different states.For a general case where MAC = *n*, assume “*n*” LRS cells connected to *V*_read_, while the remaining cells are in HRS (*m*) or not connected to *V*_read_ (*N* – *m* – *n*), then the total BL/SL current is
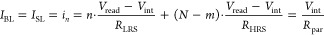
1.1

The condition for ADC to be able to distinguish between the MAC outputs provides us with
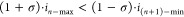
1.2where σ is the percentage variation around the mean value (Figure 2b). Parasitic resistance on the readout path (*R*_par_), appears in eqs 1.1 and 1.2, highlighting the influence of circuit parasitic on accurate readout. If the resistance ratios are represented by , the inequality leads us to
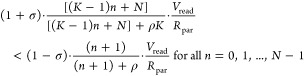
1.3

We represent the ratio of variabilities as . It can be proven that the quadratic inequality (eq 1.3) always holds true if and only if both *n* = 0 and *n* = *N* – 1 hold true. For *n* = 0, it gives
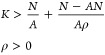
1.4

An intuitive finding from (eq 1.4) is that , which means the  cannot be smaller than the number of wordlines turned on simultaneously. For *n* = *N* – 1 (eq 1.3) gives the following:
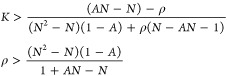
1.5

Combining eqs 1.4 and 1.5, we have
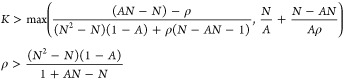
1.6

Note that the denominator in the ρ-inequality in eq 1.5 or eq 1.6 needs to be positive, and this gives a lower bound on the ratio of variabilities, *A*, i.e., an upper bound on the variability, σ:
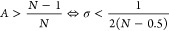
1.7

**Figure 3:**
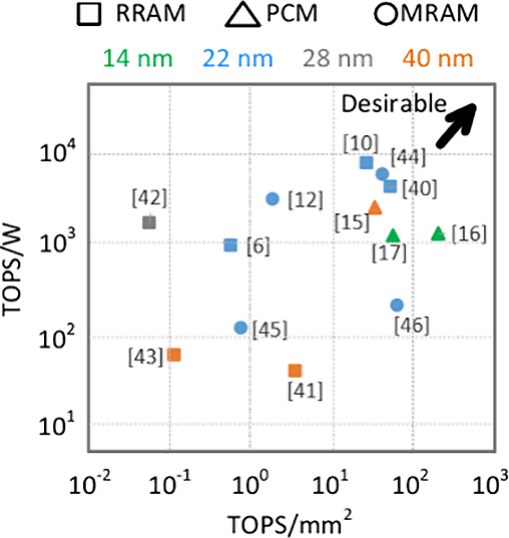
Compute density (TOPS/mm^2^) vs energy efficiency (TOPS/W) for circuit-level demonstrations of the NVM-CIM.

Figure 3c shows the constraints on  and  defined by eq 1.6. The figure reveals that large  is desirable for accurate readout. Similarly, *R*_LRS_ cannot be made arbitrarily small as this causes *R*_par_ to dominate in the current calculation and the effect of HRS/LRS on *I*_BL_ diminishes. This makes it difficult to distinguish between the MAC digital outputs. *R*_par_ is dependent on the length of the BL/SL, which is determined by size of the NVM array, metal layer in the CMOS stack as well the CMOS technology node. Therefore, the circuit level design target determines the allowed device characteristics and vice versa. Our analysis uses the resistance ratio () to make it applicable to circuit designs irrespective of technology node. As the number of WL being activated (*N*) increases, the constraints become more stringent, as intuitively expected. This is also experimentally confirmed by previous work,^30^ where CIM readout accuracy can be controlled by dynamically choosing the number of WL used in MAC computation.

The same analysis is extended to assess the effect of resistance variability (σ) in Figure 3d. Variability causes the distribution of *I*_BL_ from Figure 3b to get wider, causing the distributions to overlap. Therefore, variability has been shown to cause more stringent constraints on resistance values. Overall, it can be concluded that large *R*_LRS_ and  are desirable from the point of view of accuracy.

### Minimizing Energy-Delay Product

Arbitrarily large *R*_LRS_ causes long latency in charging up the BL capacitance, slowing down current buildup at the ADC input. Circuit level solutions to mitigate the effect of high R_LRS_ have been shown to be needed for circuit performance improvement.^31^ Therefore, besides the accuracy-oriented bound discussed earlier, the target circuit performance also constrains the range of the *R*_LRS_ and . We use energy-delay-product (EDP) metric which is widely used to incorporate the energy vs latency trade-off in computing circuits.^32,33^

We estimate average steady-state power consumption over a single BL/SL assuming 50% sparsity with half of the NVM cells being in LRS and the other half in HRS. The inputs are also assumed to have 50% sparsity as commonly used in CIM circuit benchmarks.^11,30^ This results in *N*/4 of the cells passing *I*_LRS_ and *N*/4 causing *I*_HRS_, whereas the remaining cells remain inactive. *I*_BL_ in this case corresponds to
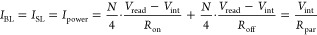
2.1And the total column-level power consumption without the ADC is
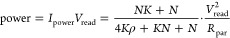
2.2

We estimate computation latency where worst case latency of BL charging is observed when only one cell operating in LRS passes current through it, causing the smallest current for *C*_par_ charge up. The latency is controlled by the RC time constant provided by
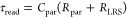
2.3

Therefore, the energy-delay-product proportionality equation is given by eq 2.4. This provides bounds on device resistances.
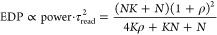
2.4

Figure 3e shows the implication of EDP constraint on resistance ratio and *R*_LRS_. *R*_LRS_ cannot be made arbitrarily high to ensure low latency readout, which eventually affects the EDP. If a larger value of EDP is allowed at the circuit level, the permissible range of *R*_LRS_ is wider assuming fixed parasitics (*R*_par_, *C*_par_). EDP does not constrain the resistance ratio strongly.

Combining the accuracy and EDP constraints leads us to the design space for designing HRS/LRS resistance values for a NVM device. The lower bound of  is determined by the accuracy constraint. The lower bound of *R*_LRS_ is determined by accuracy, while the upper bound is fixed by EDP. Our analysis proposes a quick way of assessing the design space, and a more detailed circuit-level simulation analysis is needed to incorporate more accurate distributed parasitic models. This reveals how early identification of circuit level targets and device engineering constraints may help in enabling co-optimized CIM systems.

Though this paper proposes a stringent accuracy boundary for device parameters, researchers can define their own accuracy boundary, which is more relaxed. For example, in some machine-learning applications, a certain amount of error from CIM can be tolerated. Also, various design methods can boost the computation accuracy, such as error-aware-training, ECC circuits,^34^ and voltage clamping circuits.^29^ These relax the constraints on devices and thus lead to a larger design space. In other words, the accuracy boundary in Figure 2f can move from the red solid line (*I*_BL_-separable) to the red dashed line (application-tolerated).

The analysis in this section does not assume any specific NVM device. Therefore, apart from commonly used RRAM, PCM, and MRAM, it is applicable to the ferroelectric device (FeRAM,^35^ FeFET,^36^ floating gate FET,^37^ etc.). Thus, design space for any device capable of showing 2 distinct resistance states on readout can be evaluated using this approach. Additionally, the analysis using 2 resistance levels can be extended to multilevel cells by reformulating eq 1.2 and eq 2.4.

## Write Challenges for NVM-CIM

(a) **Programming Voltages and Latencies**: Long programming latency greater than a few microseconds and voltages more than the nominal voltage of CMOS logic circuits^7^ are typically needed for changing resistance states of NVM devices.^38^ This requires additional circuit components like charge pumps^39^ which degrades the overall system efficiency. Additionally, programming is often carried out using a closed-loop write-verify scheme^40,41^ causing long programming latency for these chips before they are deployed in the field. Thus, exploration in CMOS compatible materials with lower programming resource requirement would benefit the circuit efficiency.^42^

(b) **Thermal Drift**: Continuous operation of NVM-CIM has been observed to cause gradual change in resistance even when the programming voltage is not applied.^41,43^ Circuit level implication of this phenomenon results in continuous resistance state checking circuits^41^ followed by reprogramming. TSMC’s university collaborations have demonstrated circuits to monitor such drift which may slow down the operation.^41^

(c) **Endurance**: NVM devices typically allow lower number of write cycles before they fail compared to SRAM.^7,38^ This results in NVMs failing before the CMOS logic counterparts with the chip becoming nonoperational before its actual lifetime.

Therefore, devices with stable and low voltage writing, better endurance and prevention of thermal drift would be of great benefit for field application of NVM-CIM chips.

## CIM Circuits Benchmark

We survey recent NVM-CIM demonstrations at leading circuits venues to highlight the recent advances and summarize ongoing research trends in RRAM,^5,9,44−47^ MRAM,^11,48−50^ and PCM.^14−16^Figure 3 compares the energy efficiency (TOPS/W) and compute density (TOPS/mm^2^) for several such chips. Scaling to smaller technology nodes can be generally seen to improve performance. Therefore, achieving scaling to advanced CMOS nodes is desirable. The performance of MRAMs can be seen to lag behind PCM and RRAM which is speculated to be because of their low resistance ratio (K). This limits the number of WL that can be turned on, and further limits throughput and hampers performance. Additionally, some of these chips^15,50,^ show low accuracy (>5% degradation) compared to other works for CIFAR-10 data set. This may be attributed to device parameters being in the region not permitted by accuracy constraints (Figure 2c,d). Circuit-aware device design approaches such as the one outlined in this work may be of benefit in mitigating this challenge.

## Conclusion

We analyze the constraints on resistance of NVM devices from the point of view of accuracy and EDP to identify the bounds. High resistance ratio (*K* = *R*_HRS_/*R*_LRS_) is preferred for high accuracy and low EDP. However, the absolute value of *R*_LRS_ has an upper bound for high accuracy readout and a lower bound for ensuring low EDP. The analysis is independent of the CMOS technology node, and absolute values can be easily calculated by estimating the parasitic resistance in the readout path. We also review the write challenges to advocate for the engineering effort for low voltage write, thermal stability of resistance values, and high endurance operation. Our survey of CIM circuits reveals the desirability of technology scaling for NVM devices for area and energy efficiency.
